# Relationship of atopic dermatitis with stroke and myocardial infarction: A meta-analysis

**DOI:** 10.1097/MD.0000000000013512

**Published:** 2018-12-10

**Authors:** Min Yuan, Wen-Feng Cao, Xu-Fang Xie, Huang-Yan Zhou, Xiao-Mu Wu

**Affiliations:** a Department of Neurology, Jiangxi Provincial People's Hospital; b Department of Blood Transfusion, Jiangxi Provincial Tumor Hospital, Nanchang, Jiangxi Province, China.

**Keywords:** atopic dermatitis, meta-analysis, myocardial infarction, stroke

## Abstract

**Background:**

Lots of previous reports have suggested a potential association of atopic dermatitis (AD) with stroke and myocardial infarction (MI). However, the result is still controversial, Consequently, we conducted this meta-analysis to estimate the relationship of AD with Stroke and MI.

**Methods:**

PubMed, Embase, and Web of Science databases were searched from inception to June 2018. Stroke and MI were considered as a composite endpoint. We calculated pooled hazard ratios (HRs) with 95% confidence intervals (CIs). Subgroup and sensitivity analysis were performed to assess the potential sources of heterogeneity of the pooled estimation.

**Results:**

A total of 12 articles with 15 studies involving 3,701,199 participants were included in this meta-analysis. Of these, 14 studies on stroke and 12 on MI. Pooled analysis showed participants with AD experienced a significant increased risk of stroke (combined HR, 1.15; 95% CI, 1.08–1.22; *P* = .000) and MI (combined HR, 1.13; 95% CI, 1.02–1.24; *P* = .014), compared with participants without AD. The risk of stroke and MI was significant both in male subjects (stroke: HR: 1.33, 95% CI: 1.14–1.56; MI: HR: 2.01, 95% CI: 1.31–3.08), but not in female subjects (HR: 1.02, 95% CI: 0.77–1.35; MI: HR: 0.98, 95% CI: 0.72–1.32). The results were more pronounced for ischemic stroke (HR: 1.16, 95% CI: 1.13–1.19) in the stratified with stroke type. Stratifying by AD type, the risk of stroke was significant in severe AD (HR: 1.29, 95% CI: 1.08–1.54) and moderate AD (HR: 1.11, 95% CI: 1.01–1.22) for MI.

**Conclusions:**

AD is independently associated with an increased risk of stroke and MI, especially in male subjects and ischemic stroke and the risk is associated with the severity of AD.

## 1 Introduction

Atopic dermatitis (AD) or atopic eczema is a chronic relapsing inflammatory skin disease which is characterized by drastic pruritus and eczema affecting both children and adults.^[1]^ Incidence of AD has increased 2- to 3-fold in industrialized nations, impacting approximately 15% to 20% of children and 1% to 3% of adults worldwide.^[2]^ It has become the leading non-fatal burden to health attributable to skin diseases, and may increase the risk of the immune-mediated inflammatory diseases such as asthma and allergic rhinitis.^[3]^ This creates a major public health issue.

As everyone knows, cardiovascular disease (CVD) has become a major public health issue and is responsible for approximately 30% of all deaths worldwide.^[4]^ Stroke and ischemic heart disease reminds a leading cause of death and a major cause of adult disability worldwide.^[5]^ Traditional factors such as hypertension, diabetes, hypercholesterolemia, cigarette smoking, and low level of physical activity increase the risk of stroke and myocardial infarction (MI).^[6,7]^ Nowadays, it is generally accepted that atherosclerosis is a chronic systemic inflammation disease, which is the important reason of several serious adverse events, including coronary artery disease, MI, stroke, and peripheral artery disease.^[8]^ And research shows that many skin disorders are closely associated with stroke and MI.^[9–12]^ It leads to a hypothesis that patients with AD may have an increase risk of stroke and MI, similar to inflammatory skin disorders.

During the past decade, research has suggested that AD is an allergic disease in which systemic inflammation involves more than just the skin, lots of epidemiologic observational studies have investigated the associations between AD and future stroke and/or MI.^[13–24]^ However, the results are inconsistent. Some of these researches suggested that AD may increase the risk of stroke and/or MI incidence.^[15,19,21,24]^ While several studies found that AD was not independently associated with increased risk of stroke and MI.^[13,16,18]^ Furthermore, the different results of various studies showed that stroke type, sex, and severity of AD may affect the risk of stroke and/or MI independently.^[15,18,19,21,22]^ Given these inconsistent results, to obtain a more comprehensive estimate of the putative influence of AD on stroke and MI, we conducted a meta-analysis to assess the association of AD with stroke and MI risk.

## 2 Materials and Methods

### 2.1 Ethics statement

As all analyses in our article were based on previously published studies, no ethical approval or patient consent was required.

### 2.2 Literature search

The search was conducted according to the recommendations of Preferred Reporting Items for Systematic Reviews and Meta-Analyses (PRISMA) Statement.^[25]^ A systematic search of PubMed, EMBASE, and Web of Science databases was performed up to June 2018. The following key words were used in our search: “atopic dermatitis,” “atopic eczema,” “dermatitis” or “eczema” and “stroke,” “cerebrovascular diseases” or “cerebrovascular disorders,” and “myocardial infarction,” “coronary heart disease,” “ischemic heart disease.” Studies without language and race restrictions were included to avoid publication bias. Only human studies were included. Additionally, more articles were detected through a manual search of the references from retrieved publications and recent reviews. When there were several included studies from the same or similar participants data source, only the most recent study was chosen.

### 2.3 Selection criteria

The following criteria were used in order to include the eligible studies: the study of adult patients had a cohort studies, case-control or cross-sectional design; AD was the exposure, stroke and/or MI was the outcome measure. Participants were free of stroke or MI at study entry; studies must report quantitative estimates of multivariate-adjusted odd ratio (OR) or relative risk (RR) or hazard ratio (HR) with 95% confidence interval (CI) for stroke and/or MI incidence or provided data for their calculation.

### 2.4 Data abstraction and quality assessment

Two authors (MY, XFX) independently extracted all data using a standardized data collection form. The discrepancies in data extraction were dealt with by consensus. We extracted the following data from each study: the first author's last name, year of publication, design of study, location of the participants, data source, number of participants, follow-up, exposure assessment, outcome assessment, and adjusted covariates. The Newcastle-Ottawa Scale (NOS) was used to evaluate the quality of the studies.^[26]^ The quality of the cohort study and case-control study was evaluated by the following 3 major components: the selection of the participants, the comparability between the groups and the ascertainment of the exposure. The modified NOS conducted by Herzog et al^[27]^ was used for cross-sectional study. Scores of 1 to 3, 4 to 6, and 7 to 9 were considered to be poor-, fair-, and good-quality studies, respectively.

### 2.5 Statistical analysis

The odd ratios (OR) and relative risk (RR) were considered equivalent to HRs. Stata 12 (Stata Corp, College Station, TX) was used to estimate the pooled HR and 95% CI of the association between AD with stroke and MI on the basis of a random-effects model meta-analysis. When a study reported the risk estimates adjusted for different covariates, we used the most full adjusted models in the analysis of the pooled HR. We converted HR and 95% CI into Napierian logarithms, and then we calculated the standard error (SE) from these logarithmic numbers and corresponding 95% CI.

Two methods were used to measure heterogeneity of HR across studies in this meta-analysis. The chi-square test based on Cochran *Q* test statistic was used to examine the null hypothesis that the retrieved publications were evaluating the same effect at *P* < .10 level of significance, and *I*^2^ test statistic was used to quantify heterogeneity, which grades the inconsistency in these studies’ results. *I*^2^ values of 25, 50, and 75% was regarded as low, moderate, and high, respectively. Potential publication bias was assessed by the symmetry of the funnel plot,^[28]^ as well as the Egger test, and Begg test.^[29,30]^

If there was evidence of heterogeneity, subgroup analyses and sensitivity analyses were employed to explain what contributed to the heterogeneity. Subgroup analyses based on adjusted HRs were conducted according to study type (cohort study vs cross-sectional study), sex (female vs male), geographic area (Asia vs non-Asia), AD type (mild, moderate, and severe), and stroke type (ischemic stroke vs hemorrhagic stroke). A sensitivity analysis was performed by removing each individual study from the meta-analysis to assess the possible reason for heterogeneity. In all statistic analysis, *P* values were 2-sided and *P* < .05 was regarded as statistically significant.

## 3 Results

### 3.1 Literature search and characteristics

**Figure 1:**
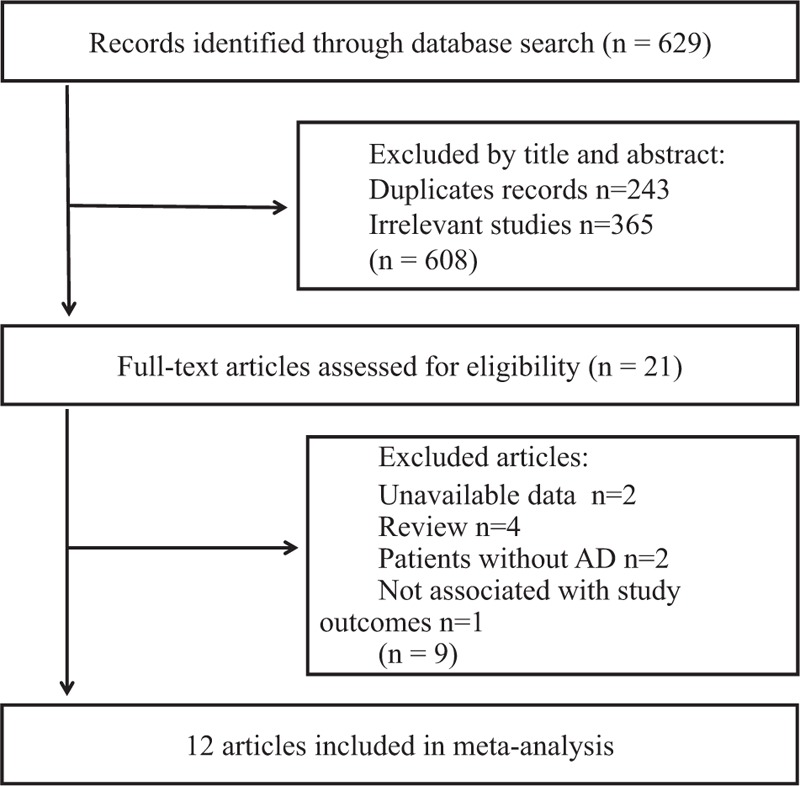
Flow chart of study selection.

A total of 629 articles were retrieved from the initial PubMed, Embase, Web of Science electronic database search. Of which 608 articles were excluded after the first screening based on titles and abstracts, leaving 21 articles for full-text review. Of these, 9 articles were excluded because data were not available in 2 publications, the articles were review in 4 publications, and the patients without AD in 2 publications, the outcome of interest was not stroke or MI in 1 publications. Finally, 12 articles were included in our meta-analysis.^[13–24]^ The results of the study selection process are shown in Fig. 1.

The characteristics of the 12 retrieved articles with 15 studies were presented in Table 1. All studies were published between 2014 and 2018 with a follow-up ranging from 1 to 18 years, except for 1 study,^[13]^ no specific follow-up time was indicated. Among these, 1 article had 2 studies,^[14]^ and 1 article had 3 studies.^[17]^ One study was conducted in the UK,^[22]^ 4 in Asia country (Taiwan),^[19–21,24]^ 3 in Demark,^[15,18,23]^ 4 in United States,^[16,17]^ 2 in Germany,^[14]^ and 1 in Canada.^[13]^ Among these, 5 were cross-sectional studies,^[13,14,17]^ 10 were cohort studies.^[14–16,18–24]^ Three focused on stroke,^[19,21,24]^ 1 focused on MI^[15]^ and 11 on both.^[13,14,16–18,20,22,23]^ All studies provided adjusted risk estimates, overall quality scores ranged from 7 to 9, and all studies were graded as good quality according to the Newcastle–Ottawa Quality Assessment Scale.

**Table 1:**
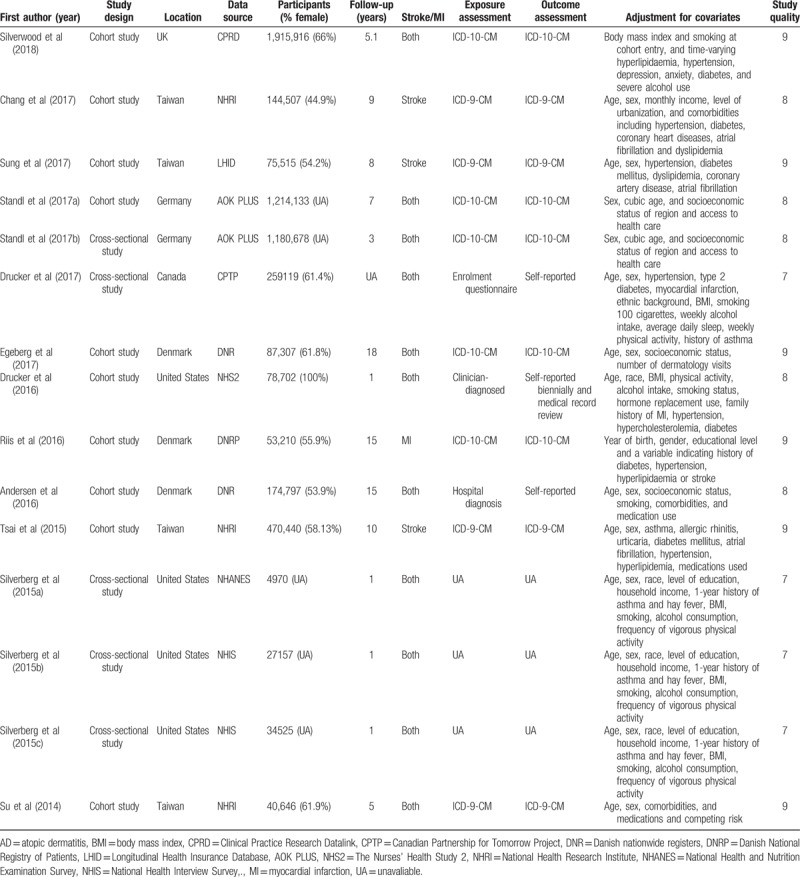
Characteristic of included studies.

### 3.2 AD and risk of stroke incidence

**Figure 2:**
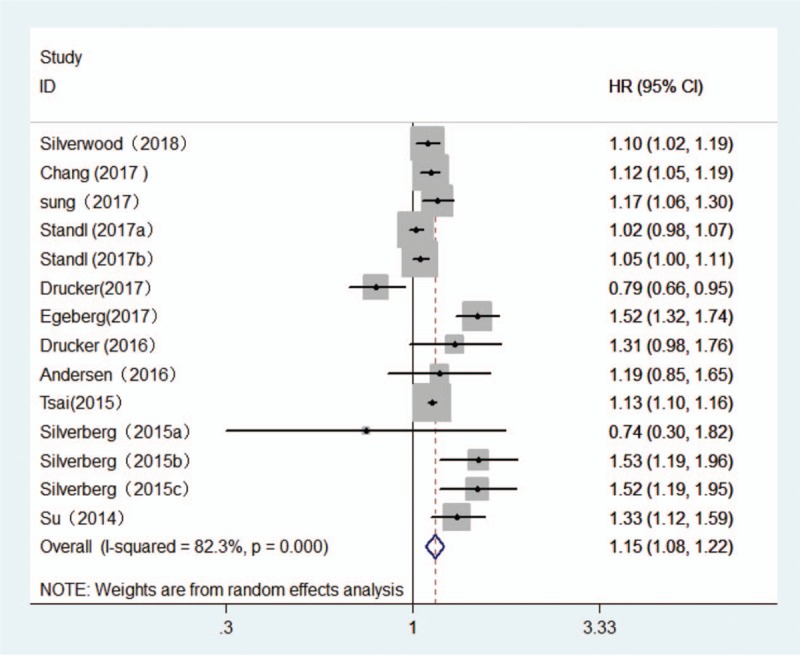
Forest plot of the association between atopic dermatitis and stroke risk.

**Figure 3:**
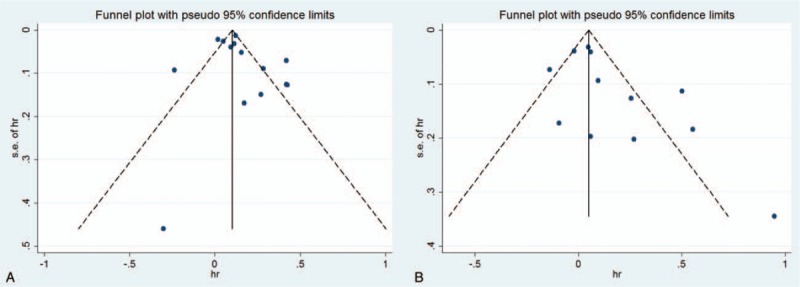
Funnel plots of atopic dermatitis and risk of stroke (A) and myocardial infarction (B).

The multivariable adjusted HRs of stroke in relation to AD from individual studies and the combined HR are presented in Fig. 2. Participants with AD experienced a significant increased risk for development of stroke based on 11 articles with 14 studies when compared with non-AD (combined HR, 1.15; 95% CI, 1.08–1.22; *P* = .000). There was evidence of moderate heterogeneity in the magnitude of the association across studies (*P* for heterogeneity = .000, *I*^2^ = 82.3%). There was no evidence of publication bias by inspection of the funnel plot (Fig. 3A). Further Begg and Egger test results also showed no evidence of publication bias for stroke (Begg: *P* = .477; Egger: *P* = .344).

### 3.3 AD and risk of MI incidence

**Figure 4:**
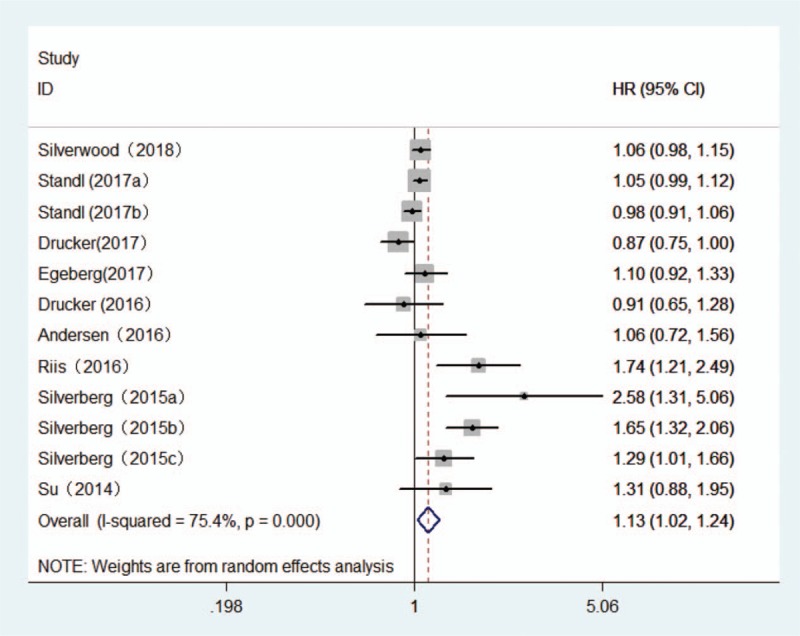
Forest plot the association between atopic dermatitis and myocardial infarction risk.

The multivariable adjusted HRs of MI in relation to AD from individual studies and the combined HR are presented in Fig. 4. Participants with AD experienced a significant increased risk for development of MI based on 9 articles with 12 studies when compared with non-AD (combined HR, 1.13; 95% CI, 1.02–1.24; *P* = .014). There was evidence of modest heterogeneity in the magnitude of the association across studies (*P* for heterogeneity = .000, *I*^2^ = 75.4%). Review of funnel plots could not eliminate the potential for publication bias for MI (Fig. 3B). While, further Begg and Egger test results showed no evidence of publication bias for MI (Begg: *P* = .10; Egger: *P* = .068).

### 3.4 Subgroup analysis

The subgroup analysis is shown in Table 2. Stratified by study type, participants with AD experienced a significant increased risk for development of stroke and MI only in cohort studies (stroke: HR: 1.16, 95% CI: 1.09–1.24, n = 9; MI: HR: 1.08, 95% CI: 1.00–1.07, n = 7), no association in cross-sectional studies (stroke: HR: 1.13, 95% CI: 0.89–1.43, n = 5; MI: HR: 1.23, 95% CI: 0.96–1.56, n = 5). After stratification by sex, AD was observed to significantly elevate the risk of stroke and MI in men (stroke: HR: 1.33, 95% CI: 1.14–1.56; n = 3; MI: HR: 2.01, 95% CI: 1.31–3.08, n = 1), but not of the women (HR: 1.02, 95% CI: 0.77–1.35, n = 3; MI: HR: 0.98, 95% CI: 0.72–1.32, n = 2). When stratified by geographic area, we found patients with AD was associated with risk of stroke both in Asia and non-Asia, while the increased risk of MI just found in non-Asia (HR: 1.12, 95% CI: 1.02–1.24, n = 11). Previous studies have shown that the stroke and MI risk probably increased with AD severity. Then we pooled the logarithm of HR for comparable categories of AD levels. As shown in Table 2, AD was associated with an increased risk of subsequent stroke in severe AD (HR: 1.29, 95% CI: 1.08–1.54, n = 3), no association was observed in mild and moderate AD. While the risk for MI was only found in moderate AD (HR: 1.11, 95% CI: 1.01–1.22, n = 1). In addition, we found participants with AD experienced a significant increased risk just for ischemic stroke (HR: 1.16, 95% CI: 1.13–1.19, n = 5), not in hemorrhagic stroke (HR: 1.07, 95% CI: 0.93–1.23, n = 2).

**Table 2:**
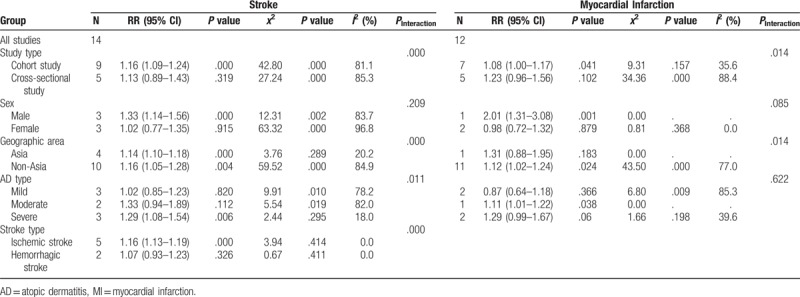
Subgroup analysis of AD with stroke and MI risk.

### 3.5 Sensitivity analysis

Sensitivity analysis was used to evaluate potential sources of heterogeneity in the association between the studies. To clear which one of the retrieved studies might influence the results, we assessed the risk estimates for the remainder of the studies by deleting 1 study at each turn. The results of the meta-analysis remained largely unchanged, indicating that the results of the present meta-analysis were stable (Fig not show).

## 4 Discussion

In this meta-analysis of 15 studies involving 3,701,199 participants, we confirmed that AD was associated with an increased risk of stroke and MI. And the risk was significant in male subjects, but not in female subjects. In addition, results indicated that this risk was more common in ischemic stroke and was associated with the severity of AD.

AD is the most common chronic relapsing and inflammatory skin disease and its pathophysiologically characterized by abnormalities of epidermal barrier function and T-cell-driven cutaneous inflammation.^[31]^ The pathogenesis of AD is attributed mostly to immune system abnormalities and hyperactivity, and mutation of several genes has been involved in the immune response, with key roles played by T-helper 2 (Th2) cell dysregulation and immunoglobulin E (IgE) production.^[32]^ In recent decades, overwhelming evidence has indicated that AD is associated with well-known risk factors for cardiovascular disease include hypertension, old age, diabetes mellitus, hyperlipidemia, and so on, while many of these are also associated with the risk of stroke and MI.^[33–36]^ There is also some information that AD may have a direct role in the development of stroke and MI.^[13–15,17,19,22]^ In addition, some believe that systemic inflammation associated with AD may increase the risk of cardiovascular and cerebrovascular diseases, similar to the increased risk of psoriasis, another chronic inflammatory skin condition.^[37,38]^ However, the potential mechanisms by which AD is independently associated with an increased of stroke and MI remains ambiguous. We speculate that patients suffering from AD may have an increased risk of cardiovascular risk similar to psoriasis which has been confirmed as an independent cardiovascular risk factor.^[39]^ However, the inflammatory pathways of AD is differ with psoriasis, that AD is mostly mediated by Th2, and psoriasis by Th1 and Th17 cytokines.^[40]^ There are several possible explanations for the high risk of stroke and MI. First, AD was associated with increased blood platelet activation, which suggested that activated platelets play a role in the pathomechanism of AD.^[41]^ Chronic inflammatory changes of the vascular wall induced by platelet result in development of atherothrombosis.^[42]^ Second, reduced fibrinolysis was detected in patients with AD, which are known to be associated with prothrombotic tendency.^[43]^ Third, oxidative stress is likely to be an important factor in the pathogenesis of AD.^[32]^ Fourth, previous studies showed that AD has been associated with several cardiovascular risk factors, including obesity, decreased physical activity, increased smoking and alcohol assumption, hypertension, hyperlipidemia, and diabetes.^[44,45]^ These mechanisms suggests that AD may be associated with a systemic effect which can lead to stroke and MI.

Over the past few decades, although the role of AD in cardiovascular or cerebrovascular diseases has been studied in previous clinical studies, it is not clear whether there is a causal link between AD with the risk of stroke and MI. Some studies suggested that AD is associated with an increased risk of stroke and/or MI incidence,^[15,19,21,24]^ while 3 studies failed to find such an association.^[13,16,18]^ A systematic review of AD and the risk of cardiovascular disease and type 2 diabetes in adults showed no association between AD and hypertension, presumed type 2 diabetes, myocardial infarction, and stroke in the quantitative crude data analyses and fully adjusted data analyses.^[46]^ But most studies were cross-sectional in this systematic review and information about sex, AD severity, and study type were lacking. In the present meta-analysis, we found that the risk of stroke and MI was higher in patients with AD and the male subjects. And similar analyses using fully cohort studies gave essentially identical results. This result is consistent with previous population-based cohort studies.^[15,19–21]^ As we all known, men have been more frequently subjected to the negative influence factors, such as smoking, drinking, and other unhealthy behaviors, which may explain why male AD patients appear to have a higher risk of stroke and/or MI than female patients. Furthermore, results indicated that this risk was more common in ischemic stroke and was associated with the severity of AD, which was consistent with the conclusions of some large sample studies.^[19–21,24]^ To our knowledge, stroke is divided into hemorrhagic stroke and ischemic stroke, although there are many vascular risk factors among the 2 major branches, but its pathophysiological basis is different, reflecting different pathogenesis. There is growing evidence to suggest that systemic inflammation can promote the progression of atherosclerosis and thrombosis to ischemic stroke,^[47,48]^ therefor, changes in atherosclerosis and activation of the coagulation system related to chronic inflammation may be one of the reasons for high risk of ischemic stroke in AD patients.^[49]^

Moderate heterogeneity across studies was observed, which did not alter much in the subgroup analyses and sensitivity analysis. Heterogeneity might have come from several sources, such as variations in characteristics of study populations, study designs, follow-up length, and adjustment for confounding factors. In the present meta-analysis, the sample size of each study, incomplete matching, country of origin, study type, and sex differences should be the main source of heterogeneity.

Several potential limitations of the current meta-analysis should be acknowledged. First, there were different clinical designs in the included studies. Most of them were cohort studies, and 3 of them were cross-sectional studies. In these included studies, history of AD was mostly self-reported and not performed clinical evaluation or affirmed through any diagnostic testing, which may lead to recall bias. Second, confounding factors for the risk of stroke or MI were not adjusted consistently in these included studies. Medications use and history of asthma or other allergic diseases may affect the association between AD with stroke and/or MI risk. Other confounders like socioeconomic status, race, and lifestyle may also influence risk of stroke and/or MI. Third, the number of studies included in the subgroup was too small. Furthermore, the assessment standards of AD and stroke or MI in the included studies were different. Finally, language bias may be another possible limitation, though, we attempted to minimize this bias by searching 3 major electronic databases with no language restriction, while, some articles published in Chinese or other non-English languages may not appear in the international journal database, resulting in incomplete search.

In conclusion, the results from this meta-analysis provide new evidence that AD is independently associated with an increased risk of stroke and MI after adjustment for established cardiovascular risk factors. Future studies on the effect of AD treatment and modifiable risk factor reduction on stroke and MI risk in AD patients are warranted.

## Author Contributions

Min Yuan and Xu-Fang Xie conceived and designed the study. Min Yuan and Wen-Feng Cao searched the databases and checked these according to the eligible criteria and exclusion criteria. Xu-Fang Xie helped develop search strategies. Xiao-Mu Wu and Huang-Yan Zhou extracted the quantitative data. Min Yuan and Wen-Feng Cao analyzed the data. Min Yuan wrote the draft of the paper. All authors contributed in writing, reviewing, or revising the paper. Min Yuan and Xu-Fang Xie were the guarantors.
